# Factors Associated with Prospective Acceptability and Preferences for Unified Transdiagnostic Cognitive-Behavioral Treatments and Group Therapy in the Portuguese General Population

**DOI:** 10.1007/s10488-024-01391-1

**Published:** 2024-06-05

**Authors:** Liliana Maria Rodrigues Pedro, Michael Fonseca de Oliveira, Marco Daniel Pereira, Ana Dias da Fonseca, Maria Cristina Canavarro

**Affiliations:** 1 Center for Research in Neuropsychology and Cognitive Behavioral Intervention (CINEICC), Faculty of Psychology and Educational Sciences, University of Coimbra, Rua do Colégio Novo, Coimbra, 3000-115 Portugal; 2 Faculty of Psychology and Educational Sciences, University of Coimbra, Coimbra, Portugal

**Keywords:** Acceptability, Preferences, Transdiagnostic cognitive-behavioral treatment, Group therapy, Unified protocol, Emotional disorders

## Abstract

Group transdiagnostic cognitive-behavioral therapy (CBT) offers a promising solution for limited mental health access in Portugal. Understanding barriers to patient adherence is crucial for successful implementation. This study aimed to characterize the prospective acceptability and preferences for unified transdiagnostic CBT and group therapy in the Portuguese general population and explore their correlates. A sample of 243 participants (18–88 years old), recruited online, completed an online survey collecting information on sociodemographic and clinical characteristics, acceptability of transdiagnostic CBT treatments, specifically of Unified Protocol (UP), acceptability of group therapy, therapeutic format preferences, beliefs about group therapy and help-seeking attitudes. Most participants were receptive to and perceived as useful both unified transdiagnostic CBT and group therapy. Overall, participants presented significantly more favorable attitudes than unfavorable attitudes toward unified transdiagnostic CBT and group therapy (*p* < .001). Multivariate analyses revealed that (1) favorable attitudes toward transdiagnostic treatments were negatively associated with being employed and positively associated with living in an urban area, and higher efficacy scores; (2) unfavorable attitudes toward transdiagnostic treatments were positively associated with being married/cohabitating and negatively associated with vulnerability scores; (3) being female, living in an urban area, and higher efficacy and myth scores emerged as positive predictors of favorable attitudes toward group therapy; and (4) efficacy and vulnerability scores and help-seeking propensity emerged as negative predictors of unfavorable attitudes toward group therapy. These findings highlight the importance of delineating strategies to increase knowledge and acceptance of unified transdiagnostic CBT and group therapy in the Portuguese population, addressing specific individual characteristics.

**Supplementary Information:**

The online version contains supplementary material available at 10.1007/s10488-024-01391-1.

The World Health Organization (2022a) reported that in 2019, 970 million people worldwide suffered from a mental illness, and only a minority of them received appropriate treatment. The COVID-19 pandemic has aggravated mental disorders in populations worldwide, particularly increasing the prevalence of emotional disorders (i.e., anxiety and depressive disorders; Santomauro et al., 2021; World Health Organization [WHO], 2022). Data from the Organisation for Economic Co-operation and Development (OECD, 2020) indicate that Portugal has the highest rates of psychological distress among European Union countries (23%). Additionally, the pandemic exacerbated an already weak mental health services infrastructure in Portugal (e.g., long waitlists, insufficient responses and geographic disparities; Entidade Reguladora da Saúde [ERS], 2023; Paulino et al., 2021). For example, according to the Portuguese health regulatory agency, in 2023, around 20,000 Portuguese individuals were waiting for a mental health consultation, with 57% classified as highly urgent (ERS, 2023).

In addition, diagnosis-specific cognitive-behavioral therapy (CBT) and individual therapy are the most commonly used treatment approaches in Portugal. Although they have demonstrable efficacy across multiple mental disorders, these approaches present some limitations, namely, failure to target comorbidities of emotional disorders and imposing high costs and burdens on both patients and health care providers (Barlow et al., 2017; Carlucci et al., 2021). Therefore, disseminating and implementing cost-effective, feasible and acceptable therapeutic approaches and formats may be the key to improving access to mental health care (OECD, 2020; WHO, 2022a).

In recent years, transdiagnostic CBT has emerged as a promising evidence-based approach that overcomes the limitations of diagnosis-specific treatments (Dalgleish et al., 2020; Newby et al., 2015; Schaeuffele et al., 2021). Unified transdiagnostic CBT treatments (e.g., Transdiagnostic Group CBT for anxiety [Norton, 2012]; Transdiagnostic Behaviour Therapy [Gros, 2014]) have been developed to address several disorders with unique CBT-based protocols, targeting common mechanisms rather than specific symptoms of each disorder (McEvoy et al., 2009; Schaeuffele et al., 2021). Thus, unified transdiagnostic CBT treatments are more efficient in treating comorbid disorders, reduce therapists’ training burden and the treatment length, and can be delivered across different adult populations (Cassiello-Robbins et al., 2020) and formats, including in group (Andersen et al., 2016; Carlucci et al., 2021; McEvoy et al., 2009) and online (Celleri & Garay 2022; Newby et al., 2016).

The Unified Protocol (UP) is a unified transdiagnostic CBT treatment developed by Barlow and his team to treat emotional disorders in adults (Barlow et al., 2018). Focusing on emotion regulation, the UP targets underlying transdiagnostic mechanisms (e.g., neuroticism, negative appraisals, experiential avoidance) through eight modules, five of which are core modules (Barlow et al., 2018, 2021; Kennedy & Barlow, 2018). The patient is expected to actively engage in the treatment process, for example, by doing homework and reading a workbook between sessions (Barlow et al., 2018). Although it is a protocolized treatment, it can be flexible according to the patient’s needs (Sauer-Zavala et al., 2017, 2019). When delivered in group, the eight modules are usually covered in 12–16 two-hour sessions, with some content adaptations, by a therapist and a co-therapist (Bullis et al., 2015; de Ornelas Maia et al., 2017; Laposa et al., 2017; Reinholt et al., 2022).

There is increasing empirical evidence of the advantages and efficacy of group-based transdiagnostic CBT (Andersen et al., 2016; Newby et al., 2015; Norton, 2012), namely the UP (Cassiello-Robbins et al., 2020; Osma et al., 2022; Reinholt et al., 2022). Compared to the individual format, the group format offers several clinical and practical advantages, including proven effectiveness for different disorders (Barkowski et al., 2020; Burlingame et al., 2003, 2013), cost-effectiveness and time efficiency (i.e., allowing therapists to treat multiple patients simultaneously), the opportunity for patients to share their difficulties with others and normalize them, group cohesion, social support and reduction of mental health stigma (Morrison, 2001; Osma et al., 2019). In addition, when the group format is combined with the online format, it offers additional advantages (e.g., increased accessibility for people with mobility difficulties; Celleri & Garay 2022; Ordem dos Psicólogos Portugueses [OPP], 2020; Schaeuffele et al., 2022). However, the group format presents some challenges. For example, one member may dominate the group; patients may find it more challenging to generalize the treatment principles to their own experiences in heterogeneous groups; patients may compare their progress to that of others, which may be discouraging; and it is more difficult to coordinate times and days for the therapeutic sessions across multiple individuals (McEvoy et al., 2009; Morrison, 2001).

Despite the empirical evidence of the efficacy of UP in group (Cassiello-Robbins et al., 2020), this protocol has not yet been implemented among Portuguese adults. However, to promote treatment adherence and the success of its implementation, it is imperative to investigate whether people will accept and be willing to use this approach and therapeutic format (Galea et al., 2022; Huynh et al., 2022; Proctor et al., 2011). According to Sidani et al. (2009), treatment acceptability is defined as an individual’s favorable attitude toward treatment approaches and modalities. It reflects how the perceived appropriateness of an intervention based on prospective or retrospective responses (Sekhon et al., 2017). Several studies have reported good retrospective acceptability of transdiagnostic CBT (Galea et al., 2022; Huynh et al., 2022; Shapiro & Gros, 2021), specifically the UP (Ametaj et al., 2021; Bentley et al., 2020; Ellard et al., 2017) and group CBT treatments (Naik et al., 2013; Okumura & Ichikura, 2014), by different stakeholders (e.g., patients and therapists). However, previous studies have focused on the acceptability of treatments after participants have experienced these types of treatments, and there is little information about the prospective acceptability of these formats, which is essential for successful implementation and patient engagement in these treatments (Sekhon et al., 2017).

Regarding patients’ preferred treatment formats, the literature shows that patients prefer individual therapy, followed by group and finally online format (Osma et al., 2019; Piper, 2008). The reasons most frequently mentioned for not preferring the group format are a lack of privacy/confidentiality, low perceived efficacy, difficulty with sharing problems in public, diminished sense of individuality, and fear of criticism and rejection (Osma et al., 2019; Piper, 2008; Shechtman & Kiezel, 2016).

Understanding the factors (e.g., sociodemographic and clinical characteristics) that are associated with the acceptability and preferences regarding unified transdiagnostic CBT and group therapy is important for identifying groups of people who are less receptive to these types of treatments and, consequently, outlining strategies to increase their receptiveness (Gonzalez et al., 2011; Magaard et al., 2017). However, the associations among these variables are still unclear. Some studies have shown that younger people have more favorable attitudes toward unified transdiagnostic CBT (Peris-Baquero et al., 2022) and group format (Strauss et al., 2015). While some studies report that women seem to have more favorable attitudes toward group format (Carter et al., 2001; Strauss et al., 2015), Liddon et al., (2018) described greater acceptability of group therapy in men. Concerning educational level, Peris-Baquero et al. (2021, 2022) showed that psychologists with higher educational levels had more favorable attitudes toward the UP in group. To our knowledge, no studies have investigated the association between residential area and receptivity to unified transdiagnostic CBT or group therapy. Regarding clinical variables, Strauss et al. (2015) found that less clinical symptomatology was associated with more favorable attitudes toward group therapy. On the other hand, Osma et al. (2019) found no differences in individuals’ sociodemographic or clinical characteristics regarding preferences for a therapeutic format.

Last, it is possible that there is an association between people’s attitudes toward unified transdiagnostic CBT or group therapy and other attitudinal variables (e.g., beliefs about group therapy, perceived stigma, psychological openness; Carter et al., 2001; Coppens et al., 2013; Magaard et al., 2017; Shechtman et al., 2010). For example, studies suggest that individuals’ expectancies about treatment efficacy are associated with treatment outcomes (Constantino et al., 2012; Sauer-Zavala et al., 2018). Considering this, it might be possible that efficacy expectancies influence willingness toward unified transdiagnostic CBT or group therapy. Vogel et al. (2007) found that perceived stigma negatively correlated with patients’ attitudes toward seeking psychological treatment, which, in turn, positively influenced receptivity to treatment. However, no studies have specifically examined the relationship between attitudes toward help-seeking and favorable attitudes toward unified transdiagnostic CBT or group therapy in the general population.

## Present Study

The present study, conducted within the Portuguese general population, aligns with the European Framework for Action on Mental Health 2021–2025, which addresses actions to overcome current mental health challenges (e.g., promoting equal access to quality healthcare and treating comorbidities effectively; WHO, 2022b). Since access to evidence-based therapies is limited in Portugal (ERS, 2023; OECD, 2020), as observed in other European countries (e.g., Spain, France, Bulgaria; Barbato et al., 2016), the implementation of unified transdiagnostic CBT in group format could optimize and reinforce the responses of mental health services (Celleri & Garay 2022). As the acceptability of interventions is crucial to their successful implementation (Sekhon et al., 2017), it is important to improve our knowledge of prospective acceptability, determinant factors (e.g., age, gender), and potential challenges (e.g., overcoming stigma). The knowledge gathered within this study will help to outline strategies for increasing individuals’ adherence to unified transdiagnostic CBT in group format (Sekhon et al., 2021; Tarrier et al., 2006).

To the best of our knowledge, no studies have explored the prospective acceptability of transdiagnostic CBT in the general population or its determinant factors, and only a few studies have examined the prospective acceptability of group therapy (e.g., Strauss et al., 2015). Therefore, in this study in the Portuguese general population, we aimed to: (1) characterize the prospective acceptability of (i.e., favorable and unfavorable attitudes towards) unified transdiagnostic CBT treatments and particularly, (2) of specific aspects of the UP in group; (3) characterize the prospective acceptability of group therapy; (4) characterize preferences regarding therapeutic format including individual, group and online formats (e.g., preference order); and (5) explore the set of sociodemographic, clinical and attitudinal (i.e., beliefs about group therapy, attitudes toward help-seeking) factors associated with favorable and unfavorable attitudes toward transdiagnostic CBT and group therapy.

## Methods

### Participants

A total of 378 participants agreed to participate in the study. Of these participants, 135 were excluded because they did not complete the survey. The final sample was composed of 243 participants, and the main sociodemographic and clinical characteristics of these participants are presented in Table 1. The participants’ mean age was 36.35 years (*SD* = 13.49, range = 18–88). Most participants were female (78.2%), were single (54.7%), had a higher education level (85.6%), were employed (67.5%), lived in an urban area (63.8%) and had an average monthly family income greater than 1000 euros (73.3%). Regarding clinical data, 19.3% of participants reported having a mental disorder, of which 87.2% were emotional disorders. Approximately 27.6% of participants were currently undergoing psychological/psychiatric treatment, but only one person was receiving therapy in the group format. Only 5.6% of participants had received group treatment in the past. The majority of participants had never received transdiagnostic treatment (95.9%) and had no knowledge of transdiagnostic treatments (62.1%).

**Table 1:** Participants’ sociodemographic, clinical characteristics and previous psychological treatment experience

	Total sample (*N* = 243)
**Sociodemographic characteristics**	
Gender, *n* (%)	
Female	190 (78.2)
Male	50 (20.6)
Other	3 (1.2)
Native language, *n* (%)	
Portuguese	241 (99.2)
Other	2 (0.8)
Marital status, *n* (%)	
Single	133 (54.7)
Married/cohabitating	97 (39.9)
Divorced/separated	11 (4.5)
Widow	2 (0.8)
Educational level, *n* (%)	
Basic education	6 (2.5)
Secondary education	29 (11.9)
Higher education	208 (85.6)
Employment status, *n* (%)	
Student	49 (20.2)
Employed	164 (67.5)
Unemployed	11 (4.5)
Retired	10 (4.1)
Other	9 (3.7)
Residence, *n* (%)	
Rural	88 (36.2)
Urban	155 (63.8)
Average monthly family income, *n* (%)	
< 1000€	47 (19.3)
≥ 1000€	178 (73.3)
No answer	18 (7.4)
**Clinical characteristics**	
Presence of a mental disorder diagnosis^a^, *n* (%)	
Yes	47 (19.3)
No	196 (80.7)
Diagnosis period, *n* (%)	
< 1 year	40 (85,1)
≥ 1 year	7 (14.9)
Current psychological/psychiatric treatment, *n* (%)	
Yes^b^	67 (27.6)
Pharmacological treatment	33 (49.3)
Individual in-person psychological treatment	36 (53.7)
Individual online psychological treatment	25 (37.3)
Group in-person psychological treatment	1 (1.5)
Group online psychological treatment	0
Other	3 (1.2)
No	176 (72.4)
**Previous psychological treatment experience**	
Psychological treatment in the past, *n* (%)	
Yes^b^	126 (51.9)
Individual in-person psychological treatment	120 (95.2)
Individual online psychological treatment	16 (12.7)
Group in-person psychological treatment	7 (5.6)
Group online psychological treatment	0
No	117 (48.1)
Knowledge of transdiagnostic treatment, *n* (%)	
Yes	86 (37.9)
No	138 (62.1)
Previous experience with transdiagnostic treatment, *n* (%)	
Yes	10 (4.1)
No	233 (95.9)

*Note.* The mean age of the participants was 36.35 years (SD = 13.49) and ranged from 18 to 88

^a^ Of the participants diagnosed with a mental disorder, 85 reported an emotional disorder

^b^ In the question “If yes, in which format?”, participants were allowed to choose more than one option

### Procedure

This cross-sectional online survey was part of a wider project conducted in Portugal that aimed to examine the acceptability, feasibility and efficacy of the UP in the group format and was approved by the Ethics Committee of the Faculty of Psychology and Educational Sciences, University of Coimbra. The inclusion criteria for participants were as follows: (a) aged 18 years or older and (b) fluent in Portuguese. Recruitment occurred through online advertisements posted on social media websites (e.g., Facebook, Instagram) and through email (snowball sampling). Data were collected between May 2022 and April 2023. The online survey was hosted Limesurvey®, and the introductory page included information about the study goals, the role of the researchers and the participants, and information regarding informed consent. Informed consent to participate in the study was obtained by selecting the option “I agree to participate in this study” before starting the questionnaire.

### Measures

#### Sociodemographic and Clinical Information

Participants’ sociodemographic (e.g., age, marital status, employment status) and clinical characteristics (e.g., current diagnosis of mental disorder, current psychological/psychiatry treatment), as well as previous psychological treatment experience (e.g., experience with transdiagnostic and group treatments, knowledge of transdiagnostic treatment), were collected through a self-report questionnaire specifically developed by the researchers for this study.

#### Anxiety and Depressive Symptoms

The Hospital Anxiety and Depression Scale (HADS; Zigmond & Snaith, 1983; Portuguese version: Pais-Ribeiro et al., 2007) was used to assess anxiety and depressive symptoms. This self-report scale is composed of 14 items organized into two subscales: Anxiety (seven items; e.g., “I feel tense or wound up”) and Depression (seven items; e.g., “I feel cheerful”). Items are rated on a 4-point scale (from 0 to 3). The total score for each subscale ranges between 0 and 21 points. According to Zigmond and Snaith (1983), a score of 11 or higher indicates the presence of clinically important anxious or depressive symptomatology. In our sample, Cronbach’s alpha values were 0.87 for the Anxiety subscale and 0.84 for the Depression subscale.

#### Acceptability of Unified Transdiagnostic CBT Treatments

The acceptability of unified transdiagnostic CBT treatments was assessed with the Transdiagnostic Psychological Treatment Acceptability Questionnaire (TPTA-Q; see Appendix 1), which was specifically developed for this study. This measure was developed based on the theoretical framework of acceptability (Sekhon et al., 2017) and the acceptability and intention-to-use survey developed by Peris-Baquero et al. (2021). A brief definition of unified transdiagnostic treatments was presented to the participants: “Transdiagnostic psychological treatments allow for the treatment of individuals with various psychological disorders (e.g., depression, anxiety) using the same protocol. They do not focus on the specific symptoms of a particular disorder but rather on the common characteristics shared across different psychological disorders”. TPTA-Q comprises 16 items rated on a 5-point Likert scale ranging from 0 (*totally disagree*) to 4 (*totally agree*). Confirmatory factor analysis supported the construct validity of the instrument TPTA-Q for assessing two dimensions: (1) Favorable Attitudes (12 items; e.g., “I would like to receive a transdiagnostic psychological treatment”) and (2) Unfavorable Attitudes (four items; e.g., “Participating in transdiagnostic psychological treatment would require effort from me”) (χ2 = 246.67, *p* < .001; CFI = 0.942; RMSEA = 0.077, 95% CI: [0.065, 0.089]). Higher scores on Favorable Attitudes indicate greater acceptance of the transdiagnostic treatment, whereas higher scores on Unfavorable Attitudes indicate lower acceptance of the transdiagnostic treatment. In our sample, Cronbach’s alpha values were 0.94 for Favorable Attitudes, and 0.76 for Unfavorable Attitudes. In addition, this questionnaire asks about participants’ interest in unified transdiagnostic CBT, the psychological problems (e.g., grief, anxiety) for which they would seek this treatment approach, and whether they think this treatment approach would be useful.

#### Acceptability of Specific Aspects of the UP in the Group Format

The Scale of Acceptability of Specific Aspects of the UP in Group was created for this study and was used to measure participants’ receptivity to the UP in the group format. It is composed of eight items (e.g., “How receptive would you be to participating in weekly group sessions?”) rated on a 5-Likert scale (from 0 = *nothing* to 4 = *extremely*). Higher scores indicate greater receptivity to specific aspects of the UP in the group format. In our sample, Cronbach’s alpha value was 0.90.

#### Acceptability of Group Therapy

The acceptability of group therapy was assessed with the Group Psychological Treatment Acceptability Questionnaire (GPTA-Q; see Appendix 2), which was specifically developed for this study. This measure was also developed based on the theoretical framework of acceptability (Sekhon et al., 2017) and the acceptability and intention-to-use survey developed by Peris-Baquero et al. (2021). GPTA-Q contains 16 items rated on a 5-point Likert scale ranging from 0 (*totally disagree*) to 4 (*totally agree*). Confirmatory factor analysis supported the construct validity of the instrument GPTA-Q for assessing two dimensions: (1) Favorable attitudes (12 items; e.g., “I believe that group psychological therapy can be effective”); (2) Unfavorable attitudes (four items; e.g., “I think group psychological treatment can have ethical or moral consequences”) (χ2 = 291.84, *p* < .001; CFI = 0.917; RMSEA = 0.087, 95% CI: [0.076, 0.099]). Higher scores on Favorable Attitudes indicate greater acceptance of the group therapy, whereas higher scores on Unfavorable Attitudes indicate lower acceptance of the group therapy. In our sample, Cronbach’s alpha values were 0.93 and 0.67 for Favorable attitudes and Unfavorable attitudes, respectively. In addition, this questionnaire asks about participants’ interest in group therapy, the psychological problems (e.g., grief, anxiety) for which they would seek this therapeutic format, and whether they think this therapeutic format would be useful.

#### Preferences Regarding Psychological Treatment Format

The Treatment Format Preferences Survey was developed for this study to assess preferences regarding treatment formats and was based on the Patient’s Psychological Format Preferences survey (Osma et al., 2019). The questionnaire asks about preferred treatment formats (i.e., “If you needed to receive psychological treatment, in which format would you prefer to receive it?”). Participants are asked to rank individual, group and online formats according to their preferences. Additionally, participants are required to select reasons for selecting these formats from a list, specifying the reasons for choosing the preferred format reasons those that supported the format’s choice in the first place (e.g., “facilitates expressing problems”) and in the third place (e.g., “lack of privacy”), with a possibility of adding other reasons. Participants are also asked if there is any format that they would never choose and, if so, to identify the reasons that they opposed that format (e.g., “being impersonal”). Last, two additional questions ask participants to identify the format they would recommend to a friend (“If a friend needed to receive psychological treatment, what format would you recommend?“) and the format that causes anxiety/discomfort (“What format of therapy would cause the most anxiety/discomfort?”).

#### Beliefs about Group Therapy

The Group Therapy Survey-Revised (GTS-R; Carter et al., 2001) was used to assess beliefs about group therapy. It comprises 25 items rated on a 5-point Likert scale ranging from 1 (*strongly disagree*) to 5 (*strongly agree*). This scale includes three subscales: 1) Efficacy (e.g., “I think group therapy is less effective than individual therapy”, 2) Myths (e.g., “I think group therapy is for people with severe problems”), and 3) Vulnerability (e.g., “I am afraid I would be criticized or humiliated by another group member”). Higher scores in each subscale are indicative of positive beliefs about group treatment. Higher Efficacy scores suggest positive expectations of the effectiveness of group therapy, whereas higher myths scores suggest fewer misconceptions of group therapy. Higher vulnerability scores indicate positive expectations about one’s ability to be vulnerable in group treatment. The original version of the instrument (Carter et al., 2001) showed good internal consistency of the three subscales (*α* = 0.78 for *efficacy*, *α* = 0.77 for *myths*, and *α* = 0.75 for *vulnerability*). In the present study, Cronbach’s alpha values ranged between 0.71 (*vulnerability* subscale) and 0.85 (*efficacy* subscale).

#### Attitudes toward Help-Seeking for Mental Health Problems

The Inventory of Attitudes Toward Seeking Mental Health Service (IATSMHS; Mackenzie et at., 2004; Portuguese version: Fonseca et al., 2017) is a self-report measure used in the present study to assess attitudes toward help-seeking for mental health problems. The IATSMHS comprises 24 items rated on a 5-point Likert scale ranging from 0 (*disagree*) to 4 (*agree*). It includes three dimensions, each consisting of eight items: *psychological openness* (e.g., “There are certain problems which should not be discussed outside of one’s immediate family”), *help-seeking propensity* (e.g., “If I were to experience psychological problems‚ I could get professional help if I wanted to”), and *indifference to stigma* (e.g., “Having a mental illness carries with it a burden of shame”). Higher scores indicate more positive attitudes toward help-seeking. In the current study, the internal consistency of the three dimensions was similar to that in the Portuguese validation study (Fonseca et al., 2017): *α* = 0.67 for *psychological openness*, α = 0.76 for *help-seeking propensity*, and *α* = 0.83 for *indifference to stigma*.

### Data Analysis

Statistical analyses were conducted using the Statistical Package for the Social Sciences (SPSS, version 27.0; IBM SPSS, Chicago, IL). Descriptive statistics were calculated to explore the sample’s sociodemographic and clinical characteristics and previous experiences and to characterize the prospective acceptability and preferences regarding unified transdiagnostic CBT treatments, specific aspects of the UP and group therapy. Comparison tests (paired-sample *t*-tests) were conducted to detect differences in characteristics between individuals with favorable and unfavorable attitudes toward both unified transdiagnostic CBT and group therapy Pearson and Spearman’s bivariate correlations, as appropriate, were used to compute the associations of sociodemographic and clinical characteristics with anxiety and depression symptoms, beliefs about group therapy, attitudes toward help-seeking and the acceptability (favorable or unfavorable) of unified transdiagnostic CBT and group therapy. Dummy variables (e.g., marital status) were coded as needed to compute the bivariate associations. According to Cohen (1988), correlation coefficients between 0.10 and 0.29 were considered to indicate weak correlations, those from 0.30 to 0.49 were considered to indicate moderate correlations, and those from 0.50 to 1.0 were considered to indicate strong correlations. Finally, four multiple linear regression analyses (one for each dependent variable) were performed to test the association of each independent variable with favorable and unfavorable attitudes toward unified transdiagnostic CBT and group therapy. Only the variables that were significantly or marginally significantly (*p* < .10) associated with the two acceptability dimensions were included in the regression models. Preliminary analysis revealed that the assumptions for multiple linear regression analysis were met. The predictors in the four models did not exhibit multicollinearity (the *tolerance* values were higher than 0.1, and the *variance inflation factor* values were lower than 10; Field, 2009).

## Results

### Prospective Acceptability of Unified Transdiagnostic CBT Treatments

Most participants expressed interest in participating in unified transdiagnostic CBT treatments (*n* = 149, 61.3%), especially to treat grief (51.9%), depression (56%), anxiety (60.9%) and trauma-related disorders (46.5%). A minority of participants answered that they would be interested in seeking unified transdiagnostic CBT treatments (4.8%) for other disorders. Additionally, unified transdiagnostic CBT treatments were recognized as “very/extremely useful” by 53.5% (*n* = 130) of participants, as “moderately useful” by 38.7% (*n* = 94) and as “not very/not at all useful” by 7.8% (*n* = 19) of participants.

Participants presented significantly more favorable attitudes (*M* = 2.78, *SD* = 0.75, range = 0.33-4) than unfavorable attitudes (*M* = 1.83, *SD* = 0.64, range = 0-3.75) toward unified transdiagnostic CBT treatments (*t*(222) = 11.76, *p* < .001, *d* = 1.21). Moreover, the sample mean scores were close to the median values of favorable and unfavorable attitudes toward unified transdiagnostic CBT treatments, indicating a moderate level of acceptability for this approach treatment.

### Prospective Acceptability of Specific Aspects of the UP in the Group Format

Table 2 shows the proportion of individuals who were or were not receptive to specific aspects of the UP in the group format. Most individuals were very or extremely receptive to using a workbook (66%), doing homework (61.8%) and filling out forms (59.8%). On the other hand, less than half of the participants were very or extremely receptive to topics specifically linked to a group format, such as sharing difficulties and experiences in the therapeutic group (31.1%) and participating in group therapy sessions led by two therapists (43.2%)

**Table 2:** Prospective acceptability of specific aspects of the UP in Group

**How receptive were people to…**	Total sample *N* (= 241)				
	Nothing *n* (%)	Not much *n* (%)	Moderate *n* (%)	Very *n* (%)	Extremely *n* (%)
1. Using a workbook	3 (1.2)	10 (4.1)	69 (28.6)	107 (44.4)	52 (21.6)
2. Doing homework	5 (2.1)	20 (8.3)	67 (27.8)	107 (44.4)	42 (17.4)
3. Filling out forms	4 (1.7)	13 (5.4)	80 (33.2)	106 (44.0)	38 (15.8)
4. Experiencing emotions in a therapeutic group	19 (7.9)	53 (22.0)	95 (39.4)	54 (22.4)	20 (8.3)
5. Doing exercises between sessions and discussing them in group	18 (7.5)	46 (19.1)	95 (39.4)	62 (25.7)	20 (8.3)
6. Sharing difficulties and experiences in the therapeutic group	16 (6.6)	51 (21.2)	99 (41.1)	56 (23.2)	19 (7.9)
7. Participating in weekly group sessions	23 (9.5)	51 (21.2)	81 (33.6)	69 (28.6)	17 (7.1)
8. Participating in group therapy sessions led by two therapists	22 (9.1)	42 (17.4)	73 (30.3)	77 (32.0)	27 (11.2)

*Note*. UP = Unified Protocol. The *N* does not add up 243 due to two missing values

### Prospective Acceptability of Group Therapy

A majority of participants expressed interest in participating in a group-based psychological treatment (*n* = 141, 58% [16% in-person, 13.2% online and 28.8% in both formats]), especially to treat grief (51.9%), depression (41.6%), anxiety (51.9%) and trauma-related disorders (48.1%). A minority of participants answered that they would be interested in seeking group therapy (6.8%) for other disorders. Moreover, 51.8% (*n* = 126) of participants perceived group therapy as “very/extremely useful”, 37.4% (*n* = 91) perceived them as “moderately useful” and 10.7% (*n* = 26) perceived them as “not very/not at all useful”.

Significant differences were found between favorable and unfavorable attitudes toward group therapy (*t*(222) = 4.37, *p* < .001, *d* = 1.21): individuals showed more favorable attitudes (*M* = 2.50, *SD* = 0.68, range = 0.25-4) than unfavorable attitudes toward group therapy (*M* = 2.14, *SD* = 0.72, range = 0.25-4). Additionally, the sample mean scores were close to the median values of favorable and unfavorable attitudes toward group therapy. This indicates a moderate level of acceptability for this therapeutic format.

### Preferences Regarding Psychological Treatment Formats

In terms of preferences for therapeutic formats (see Table 3), 81.9% of participants preferred the individual format, 9.9% preferred the group format, and 8.2% preferred the online format. The majority of participants chose the online format as their second preference (54.3%). Of the 56 participants who answered that there was a format they would never choose, only 1.8% (*n* = 1) of participants answered individual format, while 55.4% (*n* = 31) and 42.8% (*n* = 24) answered group and online format, respectively.

**Table 3:** Order of therapeutic format preferences (*N* = 243)

	Format		
	Individual, *n* (%)	Group, *n* (%)	Online, *n* (%)
First Place	199 (81.9)	24 (9.9)	20 (8.2)
Second Place	34 (14.0)	77 (31.7)	132 (54.3)
Third Place	10 (4.1)	142 (58.4)	91 (37.4)

Table 4 shows the participants’ reasons for ranking individual, group, or online formats in first and third place, as well as the reasons for rejecting those formats. To summarize, the most frequent reason for choosing the individual format was “individualized support (81.9%). Regarding the reasons why people preferred the group and online formats, the most frequent reasons provided were “sharing experiences with others” (75%) and “comfort” (80%), respectively. A “lack of privacy/confidentiality” was the most reported reason that participants either ranked group treatment in third place (62%) or rejected it (83.9%). Regarding the online format, the most frequently provided reason for ranking it in third place (65.9%) or rejecting it (79.2%) was also the same: “impersonal”.

**Table 4:** Reasons for ranking treatment format preferences

Treatment format	Reasons to choose it in the first place	Reasons to choose it in the third place	Reasons to reject it
Individual	Individualized support (*n* = 163, 81.9%); Increased privacy/confidentiality (*n* = 154, 77.4%); Facilitates the expression of problems (*n* = 132, 66.3%); Comfort (*n* = 114, 57.3%); Previous experience (*n* = 62, 31.2%); Encourages interpersonal relationships (*n* = 38, 19.1%); Efficacy (*n* = 35, 17.6%); Reduces the stigma of mental illness (*n* = 7, 3.5%); Other (*n* = 3, 1.5%); Sharing experiences with other people (*n* = 1, 0.5%)	Preference for other formats (*n* = 6; 60%); No previous experience (*n* = 2; 20%); Difficulties in interpersonal communication (*n* = 1, 10%); Impersonal (*n* = 1, 10%); Diminished sense of individuality (*n* = 1; 10%); Other (*n* = 1, 10%)	Lack of privacy/confidentiality (*n* = 1, 100%); Diminished sense of individuality (*n* = 1, 100%)
Total (*n*)	*n* = 199	*n* = 10	*n* = 1
Group	Sharing experiences with others (*n* = 18, 75%); Reduces stigma of mental illness (*n* = 13, 54.2%); Facilitates the expression of problems (*n* = 10, 41.7%); Encourages interpersonal relationships (*n* = 9, 37.5%); Comfort (*n* = 5, 20.8%); Efficacy (*n* = 4, 16.7%); Individualized support (*n* = 4, 16.7%); Previous experience (*n* = 4, 16. 7%); Increased privacy/confidentiality (*n* = 2, 8.3%); Other (*n* = 1, 4.2%)	Lack of privacy/confidentiality (*n* = 88, 62%); Difficulty expressing problems in public (*n* = 80, 56.3%); No previous experience (*n* = 61, 43%); Less control/sense of security (*n* = 51, 35.9%); Diminished sense of individuality (*n* = 49, 34.5%); Increased susceptibility to other people’s criticism (*n* = 42, 29.6%); Difficulties in interpersonal communication (*n* = 39, 27.5%); Impersonal (*n* = 37, 26.1%); Preference for other formats (*n* = 31, 21.8%); Low efficacy (*n* = 5, 3.5%); Other (*n* = 3, 2.1%)	Lack of privacy/confidentiality (*n* = 26, 83.9%); Not appropriate to personal needs and characteristics (*n* = 13; 41.9%); Difficulty expressing problems in public (*n* = 13, 41.9%); Diminished sense of individuality (*n* = 11; 35.5%); Lack of control/sense of security (*n* = 11; 35.5%); Impersonal (*n* = 8; 25.8%); Increased susceptibility to other people’s criticism (*n* = 7; 22.6%); Inefficacy (*n* = 4, 12.9%)
Total (*n*)	*n* = 24	*n* = 142	*n* = 31
Online	Comfort (*n* = 16, 80%); Increased privacy/confidentiality (*n* = 11; 55%); Facilitates the expression of problems (*n* = 10, 50%); Individualized support (*n* = 5, 25%); Previous experience (*n* = 5; 25%); Encourages interpersonal relationships (*n* = 3; 15%); Efficacy (*n* = 2, 10%); Sharing experiences with others (*n* = 3, 15%); Reduces the stigma of mental illness (*n* = 2, 10%); Other (*n* = 2, 10%)	Impersonal (*n* = 60, 65.9%); Difficulties in interpersonal communication (*n* = 37, 40.7%); Preference for other formats (*n* = 37, 40.7%); Low efficacy (*n* = 26, 28.6%); No previous experience (*n* = 24, 26.4%); Less control/sense of security (*n* = 16, 17.6%); Lack of privacy/confidentiality (*n* = 10, 11%); Diminished sense of individuality (*n* = 8, 8.8%); Difficulty expressing problems in public (*n* = 5, 5.5%); Increased susceptibility to other people’s criticism (*n* = 3, 3.3%); Other (*n* = 1, 1.1%)	Impersonal (*n* = 19, 79.2%); Inefficacy (*n* = 10, 41.7%); Not appropriate to personal needs and characteristics (*n* = 7; 29.2%); Lack of control/sense of security (*n* = 6; 25%); Lack of privacy/confidentiality (*n* = 2; 8.3%); Difficulty expressing problems in public (*n* = 1; 4.2%); diminished sense of individuality (*n* = 1, 4.2%)
Total (*n*)	*n* = 20	*n* = 91	*n* = 24

If a friend needed psychological treatment, 88.5% of participants (*n* = 215) would recommend individual therapy, 5.8% (*n* = 14) would recommend group therapy (*n* = 4), 3.7% (*n* = 9) would recommend online therapy, and 2.1% of participants (*n* = 5) would not recommend any therapeutic format.

Regarding the format that participants considered to be the most uncomfortable or most likely to elicit anxiety, most selected the group format (*n* = 156, 64.2%), followed by the online format (*n* = 47, 19.3%). Only 4.1% of participants (*n* = 10) selected the individual format. Finally, 12.3% of the participants answered that no format would cause them discomfort.

### Factors that Influence Favorable and Unfavorable Attitudes toward Unified Transdiagnostic CBT and Group Therapy

#### Preliminary Analyses

Table 5 presents the correlation coefficients of the relationships of sociodemographic, clinical and attitudinal variables with favorable and unfavorable attitudes toward unified transdiagnostic CBT and group therapy.

**Table 5:** Correlations between sociodemographic, clinical, previous experience with psychological treatment and attitudinal variables and favorable and unfavorable attitudes toward unified transdiagnostic CBT and group therapy

	Unified Transdiagnostic CBT			Group Therapy	
	Favorable attitudes		Unfavorable attitudes	Favorable attitudes	Unfavorable attitudes
Age	− 0.23***		0.16*	− 0.19**	0.08
Gender ^a^	0.22***		− 0.13^†^	0.30***	− 0.15*
Marital status^b^	− 0.12^†^		0.17**	− 0.08	0.03
Educational level	0.12^†^		− 0.13^†^	0.21**	− 0.08
Employment Status^c^	− 0.22***		0.15*	− 0.10	− 0.01
Residence^d^	0.13*		− 0.14*	0.13^†^	− 0.13^†^
Average monthly family income	− 0.00		− 0.05	-02	− 0.11
Diagnosis of mental disorder^e^	0.06		0.01	0.03	0.06
Current psychological/psychiatry treatment^f^	0.06		− 0.10	0.05	− 0.02
Anxiety Symptomatology (HADS scores)	− 0.00		0.13^†^	− 0.09	0.12^†^
Depression Symptomatology (HADS scores)	− 0.18**		0.22***	− 0.16*	0.17*
Previous psychological treatment experience^g^	0.16*	− 0.10		0.11	− 0.09
Efficacy (GTS-R scores)	0.49***		− 0.28***	0.76***	− 0.42***
Myths (GTS-R scores)	0.42***		− 0.33***	0.64***	− 0.39***
Vulnerability (GTS-R scores)	0.28***		− 0.34***	0.48***	− 0.48***
Psychological Openness (IATSMHS scores)	0.36***		− 0.25***	0.34***	− 0.22***
Help-seeking Propensity (IATSMHS scores)	0.34***		− 0.28***	0.33***	− 0.33***
Indifference to Stigma (IATSMHS scores)	0.28***		− 0.17*	0.21**	− 0.18**

*Note. N =* 223. CBT = Cognitive-behavioral therapy; HADS = Hospital Anxiety and Depression Scale; GTS-R = Group Therapy Survey-Revised; IATSMHS = Inventory of Attitudes Toward Seeking Mental Health Service

^a^ 0 = male, 1 = female; ^b^ 0 = other, 1 = married or cohabitating; ^c^ 0 = unemployed, student, retired or other,1: employed; ^d^ 0 = rural; 1 = urban; ^e^ 0 = no,1 = yes; ^f^ 0 = no, 1 = yes; ^g^ 0 = no, 1 = yes;

^†^*p* < .10; * *p* < .05; ** *p* < .01; *** *p* < .001

More favorable attitudes toward unified transdiagnostic CBT treatments were significantly correlated with younger age, gender (being female), employment status (being unemployed, student, retired or other), residential area (living in an urban area), less depression symptomatology, previous experience with psychological treatment and higher scores on all GTS-R and IATSMHS subscales. On the other hand, more unfavorable attitudes toward unified transdiagnostic CBT treatments were significantly correlated with higher age, marital status (married or cohabiting), employment status (employed), residential area (living in an urban area), higher depression symptomatology and lower scores on all GTS-R and IATSMHS subscales. Although these correlations were significant, their strengths were low to moderate.

Regarding group therapy, more favorable attitudes were significantly associated with younger age, female gender, a higher education level, less depression symptomatology and higher scores on all GTS-R and IATSMHS subscales. These correlations were predominantly weak, except for efficacy and myths scores, which were strongly correlated with favorable attitudes. Unfavorable attitudes were significantly associated with male gender, higher depression symptomatology and lower scores on all GTS-R and IATSMHS subscales.

#### Multivariate Analyses: Multiple Linear Regression Models

Four regression models were examined, considering the variables that were, in the preliminary analyses, significantly (or marginally significantly, at *p* < .10) associated with the favorable and unfavorable attitudes regarding unified transdiagnostic CBT treatments (see Table 6) and group therapy (see Table 7).

**Table 6:** Predictors of favorable and unfavorable attitudes toward unified transdiagnostic CBT treatments

	Favorable attitudes					Unfavorable attitudes			
	B (SE)	β	t	*p*		B (SE)	β	t	*p*
Age	-0.05 (0.04)	− 0.08	-1.15	0.253		-0.01 (0.02)	− 0.05	-0.61	0.54
Gender^a^	0.89 (1.13)	0.05	0.79	0.430		-0.24 (0.50)	− 0.03	-0.49	0.623
Marital status^b^	-0.55 (1.04)	− 0.04	-0.53	0.597		1.28 (0.45)	0.21	2.85	**0.005**
Educational Level	-1.21 (0.94)	− 0.08	-1.20	0.199		0.25 (0.40)	0.04	0.61	0.545
Employment status^c^	-2.63 (0.98)	− 0.16	-2.72	**0.007**		0.61 (0.42)	0.10	1.45	0.148
Residence^**d**^	1.55 (0.72)	0.09	2.14	**0.033**		-0.60 (0.39)	− 0.10	-1.54	0.125
Average monthly family income	–	–	–	–		–	–	–	–
Diagnosis of mental disorder^e^	–	–	–	–		–	–	–	–
Current psychological/psychiatry treatment^f^	–	–	–	–		–	–	–	–
Anxiety Symptomatology (HADS scores)	–	–	–	–		-0.04 (0.07)	− 0.06	-0.64	0.526
Depression Symptomatology (HADS scores)	-0.06 (0.13)	− 0.03	-0.48	0.635		0.13 (0.07)	0.17	1.76	0.080
Previous psychological treatment experience^g^	-0.34 (0.96)	− 0.02	-0.35	0.721		–	–	–	–
Efficacy (GTS-R scores)	4.45 (1.05)	0.35	4.24	**< 0.001**		0.03 (0.45)	0.01	0.06	0.953
Myths (GTS-R scores)	1.11 (1.08)	0.09	1.02	0.308		-0.62 (0.47)	− 0.13	-1.32	0.187
Vulnerability (GTS-R scores)	-0.65 (0.86)	− 0.05	-0.76	0.448		-1.18 (0.37)	− 0.25	-3.19	**0.002**
Psychological Openness (IATSMHS scores)	0.11 (0.10)	0.08	1.08	0.277		-0.02 (0.04)	− 0.04	-0.48	0.633
Help-seeking Propensity (IATSMHS scores)	0.21 (0.12)	0.12	1.73	0.086		-0.10 (0.05)	− 0.14	-1.89	0.060
Indifference to Stigma (IATSMHS scores)	0.11 (0.10)	0.08	1.15	0.250		0.04 (0.04)	0.07	0.87	0.388

*Note.* HADS = Hospital Anxiety and Depression Scale; GTS-R = Group Therapy Survey-Revised; IATSMHS = Inventory of Attitudes Toward Seeking Mental Health Service. ^a^ 0 = male, 1 = female; ^b^ 0 = other, 1 = married or cohabitating; ^c^ 0 = unemployed, student, retired or other,1: employed; ^d^ 0 = rural; 1 = urban; ^e^ 0 = no,1 = yes; ^f^ 0 = no, 1 = yes; ^g^ 0 = no, 1 = yes

The first regression model (see Table 6) was significant and explained 37% of the variance in favorable attitudes toward unified transdiagnostic CBT treatments (*F*(14, 208) = 8.72, *p* < .001). Employment status, residential area, and efficacy (GTS-R scores) were associated with favorable attitudes. Specifically, being unemployed, student, retired or other, living in an urban area and having positive expectations for the efficacy of group therapy were associated with more favorable attitudes toward unified transdiagnostic CBT treatments.

The model constructed to examine the correlates of unfavorable attitudes toward unified transdiagnostic CBT treatments (see Table 6) was also significant (*F*(14, 208) = 4.86, *p* < .001), with marital status and vulnerability (GTS-R scores) emerging as significant correlates. Being married/cohabiting and having fewer positive expectations regarding the ability to be vulnerable in group treatment were significantly associated with more unfavorable attitudes toward unified transdiagnostic CBT treatments and accounted for 24.6% of the variance.

Regarding favorable attitudes toward group therapy (see Table 7), the results indicated that gender, residential area, efficacy (GTS-R scores) and myths (GTS-R scores) were significant correlates. Being female, living in an urban area, having more positive expectations regarding efficacy, and having fewer misconceptions of group therapy were associated with more favorable attitudes toward group therapy. The regression model was significant (*F*(11, 211) = 33.64, *p* < .001) and explained 63.7% of the variance.

**Table 7:** Predictors of favorable and unfavorable attitudes toward group therapy

	Favorable attitudes					Unfavorable attitudes			
	B (SE)	β	t	*p*		B (SE)	β	t	*p*
Age	-0.03 (4.97)	− 0.05	-1.20	0.231		–	–	–	–
Gender^a^	2.57 (0.90)	0.13	2.85	**0.005**		-0.15 (0.44)	− 0.02	-0.33	0.739
Marital status^b^	–	–	–	–		–	–	–	–
Educational Level	-0.99 (0.75)	− 0.06	-1.33	0.185		–	–	–	–
Employment status^c^	–	–	–	–		–	–	–	–
Residence^d^	1.55 (0.72)	0.09	2.14	**0.033**		-0.42 (0.35)	− 0.07	-1.22	0.223
Average monthly family income	–	–	–	–		–	–	–	–
Diagnosis of mental disorder^e^	–	–	–	–		–	–	–	–
Current psychological/psychiatry treatment^f^	–	–	–	–		–	–	–	–
Anxiety Symptomatology (HADS scores)	–	–	–	–		-0.02 (0.05)	− 0.03	-0.36	0.723
Depression Symptomatology (HADS scores)	-0.07 (0.10)	− 0.04	-0.77	0.440		0.03 (0.06)	0.04	0.46	0.646
Previous psychological treatment experience^g^	–	–	–	–		–	–	–	–
Efficacy (GTS-R scores)	7.72 (0.84)	0.57	9.24	**< 0.001**		-0.83 (0.40)	− 0.18	-2.07	**0.040**
Myths (GTS-R scores)	2.45 (0.86)	0.18	2.84	**0.005**		-0.33 (0.41)	− 0.07	-0.81	0.421
Vulnerability (GTS-R scores)	1.07 (0.68)	0.08	1.58	0.116		-1.55 (0.33)	− 0.34	-4.74	**< 0.001**
Psychological Openness (IATSMHS scores)	-0.10 (0.08)	− 0.07	-1.22	0.224		0.04 (0.04)	0.08	1.06	0.289
Help-seeking Propensity (IATSMHS scores)	0.13 (0.10)	0.07	1.37	0.173		-0.14 (0.05)	− 0.21	-2.98	**0.003**
Indifference to Stigma (IATSMHS scores)	-0.07 (0.08)	− 0.05	-0.94	0.350		-0.03 (0.04)	0.07	0.92	0.358

*Note.* HADS = Hospital Anxiety and Depression Scale; GTS-R = Group Therapy Survey-Revised; IATSMHS = Inventory of Attitudes Toward Seeking Mental Health Service. ^a^ 0 = male, 1 = female; ^b^ 0 = other, 1 = married or cohabitating; ^c^ 0 = unemployed, student, retired or other,1: employed; ^d^ 0 = rural; 1 = urban; ^e^ 0 = no,1 = yes; ^f^ 0 = no, 1 = yes; ^g^ 0 = no, 1 = yes

Finally, efficacy (GTS-R scores), vulnerability (GTS-R scores) and help-seeking propensity (IATSMHS scores) emerged as significant correlates of unfavorable attitudes toward group therapy (see Table 7). Having fewer positive expectations regarding the efficacy of group therapy, having fewer positive expectations regarding the ability to be vulnerable in group treatment and having a lower help-seeking propensity were associated with more unfavorable attitudes toward group therapy. The regression model was also significant (*F*(10, 212) = 9.96, *p* < .001) and explained 32% of the variance.

## Discussion

To the best of our knowledge, this is the first study to assess the prospective acceptability and preferences of the Portuguese general population regarding both unified transdiagnostic CBT, specifically involving the UP, and group therapy, as well as to explore the factors associated with their acceptability. The main findings of the present study suggest that our sample considered unified transdiagnostic CBT and group therapy to be moderately acceptable and useful, although they preferred the individual format. Moreover, our results provide evidence that some populations may demonstrate more favorable attitudes toward these types of treatments than others.

First, we explored the prospective acceptance of unified transdiagnostic CBT treatments. Our findings revealed that most individuals in our sample seem to be receptive to these treatments and believe in their utility, particularly for addressing emotional disorders, even among those without prior experience in this therapeutic approach. Furthermore, in line with Sidani et al. (2009), who defined acceptability as an individual’s favorable attitude toward treatment, we compared individuals’ attitudes toward unified transdiagnostic CBT treatments. The overall sample exhibited significantly more favorable attitudes than unfavorable attitudes toward these treatments. However, these findings only demonstrated a moderate degree of acceptability. Several studies that assessed patients’ acceptability and satisfaction with unified transdiagnostic CBT treatments after the intervention (Huynh et al., 2022; Shapiro & Gros, 2021) have shown higher levels of acceptability, which may reflect that experience with these treatments may increase the level of acceptability.

Second, we assessed how receptive people were to specific aspects of the UP. Most participants were very receptive to using a workbook, doing homework between sessions, and doing exercises throughout the treatment. A possible explanation is that these practices are also common in individual therapy, which is common in Portugal, regardless of the therapeutic model. On the other hand, participants were less receptive to topics related to the group format, such as expressing emotions and sharing difficulties in group, which seems to be a consequence of the lack of familiarity and myths about this format (Strauss et al., 2015; Osma et al., 2019). Similarly, Christensen et al. (2022) found that patients considered it harder to engage in emotional exposures in heterogeneous groups.

Third, we also explored the Portuguese general population’s prospective acceptability of group therapy. Despite lacking prior knowledge and experience, the majority of participants appear receptive to receiving treatment in a group format and acknowledge its utility, particularly for treating emotional disorders. Our sample showed significantly more favorable than unfavorable attitudes toward group therapy. However, these results, demonstrating only a moderate degree of acceptability, suggest that experience with the group format can enhance the overall level of acceptability (Huynh et al., 2022; Perich et al., 2020).

Fourth, consistent with prior studies (e.g., Osma et al., 2019, Shechtman & Kiezel, 2016), our results indicate that, if given the chance to choose, most people would prefer to receive psychological treatment in the individual format, followed by the group format and then the online format. In addition, the majority of participants stated that they would recommend the individual format to a friend. In fact, the preference for the individual format was anticipated because it is the format most frequently used in public and private mental health services in Portugal and, therefore, the format that people are most accustomed to. In line with the findings of Osma et al. (2019), we found that among the reasons for ranking a format in first place: individualized support, increased privacy/confidentiality, ease in expressing problems¸ and comfort were the most frequently cited reasons for choosing individual therapy, whereas opportunities to share experiences with others and to diminish the stigma associated with mental illness were the most frequently cited reasons to prefer group therapy. The main arguments in favor of the online format were comfort, increased privacy/confidentiality and ease of expression of problems.

Moreover, in contrast to the findings of Osma et al. (2019), the online format was the second choice of most participants, and the group format was the third choice. The group format was also rejected by more people than the online format. One possible explanation for these findings is that since the pandemic, the general population’s familiarity with the use of technology and access to the internet has greatly increased (Bin et al., 2021; Branscombe, 2020; Sixsmith et al., 2022). Additionally, the delivery of telepsychotherapy has been growing exponentially worldwide (Curreri et al., 2023; Geller et al., 2023), particularly in Portugal (OPP, 2020), which may explain the increased preference for this therapeutic format. On the other hand, the preference for an online format may be attributed to the study’s reliance on a convenience sample recruited through an online questionnaire. It would be important to clarify whether these results would be similar in samples collected face-to-face.

In our study, it should also be noted that only one participant rejected the individual intervention format; based on the reasons given, this may have resulted from a misunderstanding of the question. This finding further indicates that the individual format of psychological intervention is the most traditional in Portugal. Concerning the reasons for rejecting a therapeutic format, our results were also congruent with those of Osma et al. (2019), with the lack of confidentiality and impersonality provided as the main arguments against group and online therapy, respectively. Additionally, the majority of people considered group therapy to be the most anxiety-inducing/uncomfortable therapeutic format. These results may be attributed to the limited experience and knowledge of the Portuguese general population regarding group and online formats. Therefore, it is essential to enhance awareness and literacy of these formats, including guidelines on privacy and confidentiality (Bernard et al., 2008; Breeskin, 2011; OPP, 2017), and highlighting their advantages over individual therapy (Morrison, 2001; Piper, 2008).

Fifth, our study explored the sociodemographic, clinical and attitudinal factors associated with favorable and unfavorable attitudes toward unified transdiagnostic CBT and group therapy. Consistent with the literature (Peris-Baquero et al., 2022; Strauss et al., 2015; Wetherell et al., 2004), younger people seem to have more favorable attitudes and less unfavorable attitudes toward unified transdiagnostic CBT, as well as more favorable attitudes toward group therapy. Age differences in the acceptability of unified transdiagnostic CBT and group therapy may occur because older adults underutilize psychological treatments in general (Wetherell et al., 2004) and may have less knowledge about these specific treatment approaches and modalities. Consequently, they may be less receptive to receiving them.

Gender was also positively associated with favorable attitudes toward both unified transdiagnostic CBT and group therapy and negatively associated with unfavorable attitudes toward group therapy. Specifically, our results showed that females were more receptive to these treatments than males. These results are consistent with other studies (Nam et al., 2010; Shechtman et al., 2010; Strauss et al., 2015), but differ from the findings reported by Liddon et al. (2018), who found that men were more receptive to group therapy. One possible explanation for these findings is that women have higher mental health literacy (Hadjimina & Furnham, 2017), higher awareness of mental health disorders and greater openness to seeking psychological help (Wetherell et al., 2004), which may translate into higher acceptance of new delivery formats. In this regard, it would be crucial to better understand the reasons that older individuals and males exhibit lower acceptance of unified transdiagnostic CBT and group therapy and to define specific strategies for these groups to promote the acceptability of these treatments.

Marital status (being married or cohabiting) was significantly and positively correlated with only unfavorable attitudes toward unified transdiagnostic CBT, while a higher education level was significantly and positively correlated with only favorable attitudes toward group format. Regarding employment status, employed people seemed to have less favorable and more unfavorable attitudes toward unified transdiagnostic CBT. In contrast, there was no correlation between this sociodemographic variable and the acceptability of group format. Based on a systematic review conducted by Roberts et al. (2018), which demonstrated that people who live alone and who are not employed may have greater availability to seek mental health services, we hypothesized that people with these sociodemographic factors may be more receptive to experimenting with unified transdiagnostic CBT.

Regarding place of residence, urban residents seemed to have more favorable attitudes and fewer unfavorable attitudes toward unified transdiagnostic CBT than rural residents. Consistent with earlier research, these associations may be explained by the fact that, in general, people in rural areas are more isolated, have less access to health services and have less mental health literacy (Hayslip et al., 2010; Jones et al., 2011). Additionally, they may be more likely to feel a sense of stigmatization and refuse to engage in psychological help-seeking in general (Jones et al., 2011; Kessler et al., 2015; Shechtman et al., 2010).

In the multivariate analyses, only employment status (being unemployed, student, retired or other) and residential area (living in an urban area) emerged as significant predictors of favorable attitudes toward unified transdiagnostic CBT treatments, while marital status (being married or cohabitating) emerged as a significant predictor of unfavorable attitudes. Additionally, female gender and residential area (living in an urban area) were significant correlates of favorable attitudes toward group therapy. None of the sociodemographic factors emerged as a significant predictor of unfavorable attitudes, suggesting that other variables (e.g., beliefs about group therapy) may play a role in the development of more unfavorable attitudes toward group format.

With respect to clinical variables, less depressive symptomatology was associated with greater acceptability of both unified transdiagnostic CBT and group therapy, consistent with Strauss et al. (2015). However, this variable was not found to be significant in the regression models. There was no significant correlation between anxiety symptomatology and the study variables. One possible explanation is that depressive symptoms, such as negative affectivity; behavioral inhibition; isolation; negative judgments of oneself, others, and the world; and cognitive inflexibility, may limit a patient’s receptivity to new therapeutic formats, such as unified transdiagnostic CBT and group therapy, about which they have no prior knowledge or experience (Beck & Alford, 2009; Bullis et al., 2019; Strauss et al., 2015). Similarly, previous psychological treatment experience was not significantly associated with attitudes toward unified transdiagnostic CBT or group therapy, even though it was positively associated with favorable attitudes toward the transdiagnostic approach in the univariate analysis. Notably, only a small proportion of participants in our sample had previous experience with group treatment, so it is possible that they did not have enough knowledge to take an attitude toward group therapy.

Our results provide some innovative findings, as they suggest that attitudinal variables are possible determinants of the prospective acceptability of unified transdiagnostic CBT and group therapy. In the univariate analysis, all dimensions of beliefs about groups and all dimensions of attitudes toward help-seeking for mental health problems were significantly associated with the acceptability of these treatments. However, the four regression models demonstrated that only some of these variables seem to play an important role in the development of favorable and unfavorable attitudes toward both unified transdiagnostic CBT and group therapy.

In this context, efficacy was positively correlated with favorable attitudes toward both unified transdiagnostic CBT and group therapy and negatively correlated with unfavorable attitudes toward only group therapy. These findings suggest that individuals’ positive expectations about the efficacy of unified transdiagnostic CBT and group therapy may contribute to the acceptance of these treatments, in line with other studies showing that these expectations are associated with treatment outcomes (Constantino et al., 2012; Sauer-Zavala et al., 2018). As our sample mostly had higher education levels, it is expected that participants would have higher mental health literacy and better critical thinking skills, so their perception of the effectiveness and helpfulness of the treatment may have influenced their opinion about unified transdiagnostic CBT and group therapy (Bonabi et al., 2016). Furthermore, people who seek psychological help on their own and who do not have severe psychopathology tend to rate treatments as more effective (Alang & McAlpine, 2019). In our study, we recruited voluntary participants from the general population, and only a minority (19.3%) reported being diagnosed with a mental disorder. Regarding myths about therapy, fewer misconceptions of group therapy were associated with more favorable attitudes toward group-based psychological treatment. Positive expectations regarding the ability to be vulnerable in group treatment were also negatively correlated with unfavorable attitudes toward both unified transdiagnostic CBT and group therapy. In fact, the myths and vulnerability subscales of the GTS-R assess patients’ concerns about group therapy (e.g., lack of treatment personalization, loss of individuality, fear of criticism and rejection), which can influence patients’ receptiveness to this therapeutic format (Carter et al., 2001). Thus, our findings reinforce the importance of discussing the advantages and disadvantages of group therapy with patients to overcome possible barriers to treatment adherence (Carter et al., 2001; Piper, 2008).

Finally, concerning attitudes toward help-seeking for mental health problems, only help-seeking propensity was negatively correlated with unfavorable attitudes toward group therapy. This dimension relates to the willingness to actively seek help for psychological difficulties (Fonseca et al., 2017). In this sense, it is expected that individuals with a greater help-seeking propensity will also show greater acceptability of different therapeutic formats, including group format. However, further research is needed to explore why this association did not emerge for unified transdiagnostic CBT. Lastly, in univariate analyses, psychological openness and indifference to stigma were linked to the prospective acceptability of both unified transdiagnostic CBT and group therapy. This suggests that individuals with less stigma about mental health services and greater openness to these services are more receptive to transdiagnostic and group treatments (Shechtman et al., 2010; Vogel et al., 2007; Coppens et al., 2013). However, these variables did not significantly correlate with any dimension of attitudes about this approach and therapeutic format. These results seem to suggest that the degree of openness to help-seeking and stigma are not strong barriers to people’s receptivity to these treatments (James & Buttle, 2008; Mackenzie et al., 2006). Given the evidence that cultural context influences the development of beliefs and attitudes toward psychological help (Nam et al., 2010; Fekih-Romdhane et al., 2023), this variable may also affect receptivity to unified transdiagnostic CBT and group therapy. This hypothesis should be further investigated in future studies.

### Implications

Despite the exploratory nature of this study, our findings highlight important implications for practice, especially for the future dissemination and implementation of unified transdiagnostic CBT, particularly the UP, in group format. Although our sample was moderately receptive to these types of treatments, it is important to be aware that there are specific populations (e.g., young people, women, urban residents) who may have higher receptivity to these treatments. For this reason, it might be essential to outline strategies to increase the favorable attitudes of less receptive groups, namely, older adults, men, married people, those with less education and those living in rural areas.

First, it may be important to enhance understanding of the perspectives and beliefs of these populations (Wetherell et al., 2004), as well as the influence of cultural context (Nam et al., 2010) on their acceptance and preferences regarding unified transdiagnostic CBT in group. Second, the current study emphasized the usefulness of providing training and education on this approach and therapeutic format for these specific populations. Specifically, tailoring information based on sociodemographic characteristics (e.g., age and academic level) and cultural context is crucial (Wetherell et al., 2004). For example, it might be necessary to focus attention on disseminating information among men and to promote mental health literacy by conducting public education campaigns in clinical and nonclinical settings, especially in rural areas (Liddon et al., 2018; Strauss et al., 2015). Moreover, as misconceptions about group therapy and attitudes toward psychological help-seeking were factors associated with attitudes toward unified transdiagnostic CBT and group therapy, it is important to address these misconceptions (e.g., about group exposure), increase the propensity to seek help and clarify the benefits of these treatments for the general population, as well as to make people aware of the proven efficacy for different disorders and what to expect from these treatments. It is also essential to increase specific therapist training (e.g., understanding of group therapy processes) and demonstrate the advantages (e.g., cost-effectiveness) of unified transdiagnostic CBT and group therapy for mental health services to health care providers in terms of improving patients’ adherence and successful dissemination of this approach and therapeutic format (Bernard et al., 2008; Piper, 2008).

Finally, because the online format was the second preference of the majority of our sample, this format may be a good solution to reduce the stigma associated with individual (face-to-face) therapy and to increase access to mental health services for residents in more isolated geographical areas, as well as for people with less schedule availability or mobility (e.g., employees, older adults; Curreri et al., 2023; Schaeuffele et al., 2022). Originally developed for the face-to-face format, the UP can be adapted and implemented in the online format (Curreri et al., 2023). Additionally, providing group therapy in the online format via videoconferencing is even more cost-effective and accessible (Celleri & Garay 2022). However, to our knowledge, research on the application of the UP in an online group format has only recently begun to be conducted in Spain (Quilez-Orden et al., 2020) and Argentina (Celleri & Garay 2022).

Future studies should explore the feasibility, acceptability and effectiveness of implementing unified transdiagnostic CBT (e.g., the Unified Protocol) in the online group format. Specifically, given that people seem to be less receptive to aspects of the UP in the group format, in addition to the considerations mentioned above (e.g., additional training for therapists), it may be important to review the group manual and adapt some modules and exercises to online group formats (e.g., the logistics of reviewing worksheets in groups or preparing emotional exposures in-session to be conducted independently for homework; Cassiello-Robbins et al., 2020; Christensen et al., 2022; Curreri et al., 2023).

### Limitations and Future Directions

Although this study provides important contributions, some limitations should be acknowledged. First, given the cross-sectional design of the study, we could not establish causal relationships between the study variables. Second, our sample was self-selected and was not representative of the Portuguese population. It is possible that participants who completed the questionnaire had the most interest in this topic, which may influence their knowledge and attitudes. Moreover, the online survey was restricted to people with internet access. Additionally, our sample was small, mostly composed of women, highly educated, mostly employed and had high income levels. Therefore, caution is warranted in the generalization of our findings to men, people with lower education levels and people with lower incomes who may not have access to technology and the internet. Finally, there was a considerable number of incomplete questionnaire responses, possibly resulting from the questionnaire’s length, which reduced the statistical power of the study.

To obtain a larger and more representative sample and to minimize potential bias, future studies should include other recruitment and assessment methods, which could lead to more robust findings. Moreover, future studies with longitudinal designs are needed to verify the associations found in this exploratory study and examine other research questions, namely, the role of public education campaigns in attitudes toward transdiagnostic and group treatments, as well as the difference in receptivity to these treatments before and after their implementation.

## Electronic supplementary material

Below is the link to the electronic supplementary material.

Supplementary material 1

## Declarations

This study performed in line with the STROBE guidelines.
