# Supplementary material for: Factors Associated with Prospective Acceptability and Preferences for Unified Transdiagnostic Cognitive-Behavioral Treatments and Group Therapy in the Portuguese General Population
Source: Adm Policy Ment Health. 2024 Jun 5;51(6):857–76. doi: 10.1007/s10488-024-01391-1 (PMC11489295; doi:10.1007/s10488-024-01391-1)
Supplement: Supplementary file 1 — Supplementary material 1 [file 10488_2024_1391_MOESM1_ESM.docx]

**Appendix 1.** Transdiagnostic Psychological Treatment Acceptability Questionnaire (TPTA-Q)

Instructions: Transdiagnostic psychological treatments allow the treatment of individuals with various psychological disorders (e.g., depression, anxiety) because they do not focus on the specific symptoms of a particular disorder but on the common characteristics of different psychological disorders.

Please choose the number that best corresponds to how much you agree with each statement about transdiagnostic psychological treatments, according to the following scale: 0 = Totally disagree; 1 = Disagree; 2 = Neither agree nor disagree; 3 = Agree; 4 = Totally agree.

| ___ 1. I would like to receive a transdiagnostic psychological treatment. |
| --- |
| ___ 2. If I were diagnosed with a psychological problem (e.g., depression, anxiety), I would accept receiving transdiagnostic psychological treatment. |
| ___ 3. Participating in transdiagnostic psychological treatment would require effort from me. |
| ___ 4. I think transdiagnostic psychological treatment can have ethical or moral consequences. |
| ___ 5. Transdiagnostic psychological treatment could help me improve my (psychological) health. |
| ___ 6. I believe that transdiagnostic psychological treatment can result in improvements in different emotional disorders. |
| ___ 7. I feel that transdiagnostic psychological treatment can have a lasting effect. |
| ___ 8. I would recommend transdiagnostic psychological treatment to my friends. |
| ___ 9. If I needed psychological support, I would feel confident about participating in a transdiagnostic psychological treatment. |
| ___ 10. If my family/friends knew, I think they would support my participation in a transdiagnostic psychological treatment. |
| ___ 11. People who are important to me (e.g., partner, friends) would think it's a good idea for me to participate in a transdiagnostic psychological treatment. |
| ___ 12. It would be very difficult for me to organize my life to participate in transdiagnostic psychological treatment. |
| ___ 13. If necessary, I would have the necessary conditions to participate in transdiagnostic psychological treatment. |
| ___ 14. Participating in transdiagnostic psychological treatment would require a lot of time and energy from me. |
| ___ 15. I believe that transdiagnostic psychological treatment can be effective. |
| ___ 16. I consider it acceptable to receive transdiagnostic psychological treatment. |

**Appendix 2.** Group Psychological Treatment Acceptability Questionnaire (GPTA-Q)

Instructions: Please choose the number that best corresponds to how much you agree with each statement about group psychological treatment, according to the following scale: 0 = Totally disagree; 1 = Disagree; 2 = Neither agree nor disagree; 3 = Agree; 4 = Totally agree.

| ___ 1. I would like to receive group psychological treatment. |
| --- |
| ___ 2. If I were diagnosed with a psychological problem (e.g., depression, anxiety), I would accept receiving group psychological treatment. |
| ___ 3. Participating in group psychological treatment would require effort from me. |
| ___ 4. I think group psychological treatment can have ethical or moral consequences. |
| ___ 5. Group psychological treatment could help me improve my (psychological) health. |
| ___ 6. I believe that group psychological treatment can result in improvements in different emotional disorders. |
| ___ 7. I feel that group psychological treatment can have a lasting effect. |
| ___ 8. I would recommend group psychological treatment to my friends. |
| ___ 9. If I needed psychological support, I would feel confident about participating in group psychological treatment. |
| ___ 10. If my family/friends knew, I think they would support my participation in group psychological treatment. |
| ___ 11. People who are important to me (e.g., partner, friends) would think it's a good idea for me to participate in group psychological treatment. |
| ___ 12. It would be very difficult for me to organize my life to participate in group psychological treatment. |
| ___ 13. If necessary, I would have the necessary conditions to participate in group psychological treatment. |
| ___ 14. Participating in group psychological treatment would require a lot of time and energy from me. |
| ___ 15. I believe that group psychological treatment can be effective. |
| ___ 16. I consider it acceptable to receive group psychological treatment. |
